# History of ZIKV Infections in India and Management of Disease Outbreaks

**DOI:** 10.3389/fmicb.2018.02126

**Published:** 2018-09-12

**Authors:** Svetalana Khaiboullina, Timsy Uppal, Ekaterina Martynova, Albert Rizvanov, Manoj Baranwal, Subhash C. Verma

**Affiliations:** ^1^Department of Microbiology and Immunology, Reno School of Medicine, University of Nevada, Reno, NV, United States; ^2^Department of Exploratory Research, Scientific and Educational Center of Pharmaceutics, Kazan Federal University, Kazan, Russia; ^3^Department of Biotechnology, Thapar Institute of Engineering and Technology, Patiala, India

**Keywords:** Zika virus (ZIKV), epidemiology, emerging infections, India, ADEs, *Aedes aegypti*

## Abstract

Zika virus (ZIKV) is an emerging arbovirus infection endemic in multiple countries spread from Asia, Africa to the Americas and Europe. Previously known to cause rare and fairly benign human infections, ZIKV has become a major international public health emergency after being linked to unexpected neurological complications, that includes fetal brain damage/death and microcephaly in babies born to infected mothers and Guillain-Barre syndrome (GBS) in adults. It appears that a single genetic mutation in the ZIKV genome, likely acquired during explosive ZIKV outbreak in French Polynesia (2013), made virus causing mild disease to target fetus brain. The *Aedes* mosquitoes are found to be the main carrier of ZIKV, passing the virus to humans. Originally isolated from patients in Africa in 1954 (African lineage), virus disseminated to Southeast Asia (Asian lineage), establishing new endemic foci, including one in India. Numerous cases of ZIKV infection have been reported in several locations in India and neighboring countries like Pakistan and Bangladesh since mid of the last century, suggesting that the virus reached this part of Asia soon after it was first discovered in Uganda in 1947. Although, the exact means by which ZIKV was introduced to India remains unknown, it appears that the ZIKV strain circulating in India possibly belongs to the “Asian lineage,” which has not yet been associated with microcephaly and other neurological disorders. However, there still exists a threat that the contemporary ZIKV virulent strain from South America, carrying a mutation can return to Asia, posing a potential crisis to newborns and adult patients. Currently there is no specific vaccine or antiviral medication to combat ZIKV infection, thus, vector control and continuous monitoring of potential ZIKV exposure is essential to prevent the devastating consequences similar to the ones experienced in Brazil. However, the major obstacle faced by Indian healthcare agencies is that most cases of ZIKV infection have been reported in rural areas that lack access to rapid diagnosis of infection. In this review, we attempt to present a comprehensive analysis of what is currently known about the ZIKV infection in India and the neighboring countries.

## Introduction

Zika virus (ZIKV), is a mosquito-borne virus that belongs to *Flavivirus* genus of the *Flaviviridae* family, which includes other members, namely dengue virus (DENV), yellow fever virus (YFV), West Nile virus (WNV), Japanese encephalitis virus (JEV), and tick-borne encephalitis virus (TBEV) (Pierson and Kielian, 2013; Gatherer and Kohl, 2016). ZIKV is a positive-sense single-stranded RNA virus with an approximately 10.7 kb genome encoding 3,419 aa or a single polyprotein that is cleaved post-translationally by host and viral proteases into 3 structural proteins; i.e., capsid (C), pre-membrane (prM), and envelope (E), and 7 non-structural proteins (NS1, NS2A, NS2B, NS3, NS4A, NS4B, and NS5) (Kuno and Chang, 2007).

During the recent large outbreak in Brazil, ZIKV emerged as an infectious agent (Triunfol, 2016) affecting the fetal brain development causing severe deformities, and sometimes death (before or shortly after birth) (de Oliveira et al., 2017). It appears that ZIKV mutated during the earlier outbreak in French Polynesia, where, retrospective study revealed association between microcephaly and virus infection (Cauchemez et al., 2016). Targeting the fetus was the major feature of this epidemic, since, ZIKV or other known flaviviruses have never been previously associated with fetal abnormalities (Polonio et al., 2017; van den Pol et al., 2017). It appears that ZIKV selectively infects progenitor cells, while leaving differentiated cells unaffected (Zhu et al., 2017). However, in pregnant women, ZIKV crosses placenta and infects fetus brain, which presents as microcephaly (a smaller than normal head) in newborns (Schuler-Faccini et al., 2016; Coelho and Crovella, 2017). Also, cases of stillbirth due to ZIKV infection have been reported (Martines et al., 2016; Sarno et al., 2016; van der Eijk et al., 2016).

Zika virus infection in adults is mild and self-limiting and, often, infected individuals do not seek any medical attention (Duffy et al., 2009). Symptomatic ZIKV infection is characterized by fatigue, rash, headache, exanthema, fever, arthralgia, myalgia and conjunctivitis (Cerbino-Neto et al., 2016). Exanthema is the most prominent sign of infection in majority of the documented cases, and symptoms usually resolve within 2 weeks without post-morbid sequelae (Cerbino-Neto et al., 2016). In some patients, however, severe neurological complications are diagnosed, including meningitis, meningoencephalitis, and Guillain-Barre syndrome (GBS) (World Health Organization, 2016). GBS is rare but serious autoimmune disease targeting nerves (Cao-Lormeau et al., 2014). GBS is the most often diagnosed neurological sequelae in ZIKV-infected individuals. Interestingly, the higher incidence of GBS was diagnosed in older population, which makes this group of patients particularly vulnerable to post-ZIKV infection neurological complications.

## Modes of ZIKV Transmission

Zika virus is transmitted by *Aedes* species mosquitoes, primarily *Aedes albopictus, and Aedes aegypti*. There is a close relationship between the host and mosquito vector, where mosquitoes become infected while feeding off the host blood and pass the virus to the next host during the blood meal process (Roundy et al., 2017). Indian territory is a natural habitat for invasive *Aedes* mosquitoes which are widely distributed throughout the country (Kalra et al., 1997). The *Aedes albopictus* carrying ZIKV, is the most invasive species and spread rapidly in many countries of the Old and New Worlds (Manni et al., 2017). The unique ability of *Aedes albopictus* to propagate in the wild as well as near human settlements was essential for establishing presence of this vector in new areas (Padmanabha et al., 2012). The *Aedes albopictus*, which is ubiquitously present throughout the year in India, is however, more often associated as the vector for DENV transmission (Kumari et al., 2011). Primarily *Aedes aegypti* is screened for ZIKV in India but more studies are required to evaluate the role of other *Aedes* species currently in circulation of ZIKV in India.

In addition to *Aedes*, other mosquito species, including *Anopheles*, *Culex*, *Eretmapodites*, and *Mansonia* could also transmit the virus (Guo et al., 2016; Moghadam et al., 2016; Guedes et al., 2017). More recently, according to two controversial reports from China and Brazil, *Culex Quinquefasciatus* has been reported to transmit the ZIKV (Guo et al., 2016; Guedes et al., 2017). The *Culex Quinquefasciatus* is also widespread across India, similar to the *Aedes* species (Mukhopadhyay and Hati, 1978; Bhattacharya, 2005) and in some regions of India, up to 96% of all mosquito bites were from *Culex* (Mukhopadhyay and Hati, 1978). Like *Aedes species, Culex* mosquitoes also propagate near human dwellings and are found in overflow water from houses, ground pools, ditches and shallow wells (Bhattacharya and Basu, 2016). *Culex* is considered as an opportunistic feeder, as in addition to humans, it could also feed off pigs, cows and birds (Chakraborty et al., 1986). Therefore, the role of Culex in ZIKV transmission to human was extensively studied in laboratory and field collected mosquitoes. Results of vector competence study have demonstrated that *C quinquefasciatus* is poor ZIKV transmitter and, therefore, it is unlikely to contribute to virus epidemiology (Amraoui et al., 2016; Ferreira-de-Brito et al., 2016; Guerbois et al., 2016; Weger-Lucarelli et al., 2016). As a conclusion, *Aedes* species are accepted now as the main carriers of ZIKV, population control as well as positivity for ZIKV antigens of these two species could serve as a predictor of the future outbreaks.

It is accepted that vector control is essential for prevention of mosquito-borne infections. Elimination of the adult mosquitoes is usually not the most effective vector control approach. Instead, targeting larvae by eliminating or cleaning the water-holding reservoirs around the houses has been shown as an effective way to significantly reduce the mosquitoes population (Gubler, 1989; Reiter and Gubler, 1997). This approach was successfully used earlier to control the spread of mosquito-borne DENV infections (Kouri et al., 1998).

However, it appears that arthropod control should be maintained continuously as mosquitoes can quickly re-populate territories once the use of insecticides is discontinued. (Kouri et al., 1998). Also, mosquitoes can develop resistance to insecticides. Studies have shown that *Culex* species mosquitoes have developed resistance to commonly used insecticides that has hampered the efforts to reduce mosquito population (Pocquet et al., 2013). The resistance to a variety of insecticides may increase the risk of ZIKV spread into the new territories. Therefore, it appears that use of insecticide approach may not be the best to achieve the reduction of mosquito population. It has been suggested that community-based strategies to reduce the larval sources will be the cost-effective and more sustainable approach. This approach requires community education, where people living in areas populated by *Aedes* mosquitoes become aware of potential health hazards and have access to programs providing education on larval habitat control.

A novel, genetics-based vector control is focused on genetic modification of mosquitoes to decrease their propagation capability (recently reviewed by Macias et al., 2017). Multiple approaches were developed including mosquito gene editing using CRISPR/Cas9 (Gantz et al., 2015), homing gene drives (Windbichler et al., 2011), meiotic drive and sex distortion (Burt, 2003) and many others (recently reviewed by Hammond and Galizi, 2017). These approaches provide the most targeted and cost-effective interventions, which could be used as complementary to existing preventive measures. As much as exciting these technologies are, concerns about dissemination of genetic modifications across various species was expressed by scientific community (James and Collins, 2016). Also, potential negative impact on the natural ecosystem should be considered. The high likelihood of serious consequences requires careful approach for implementing genetics-based vector control including informed consent from governments and regulators as well as the local communities. Therefore, education of government and communities is essential for successful implementation of gene base control of mosquito.

## Historical Overview of ZIKV in India

**FIGURE 1:**
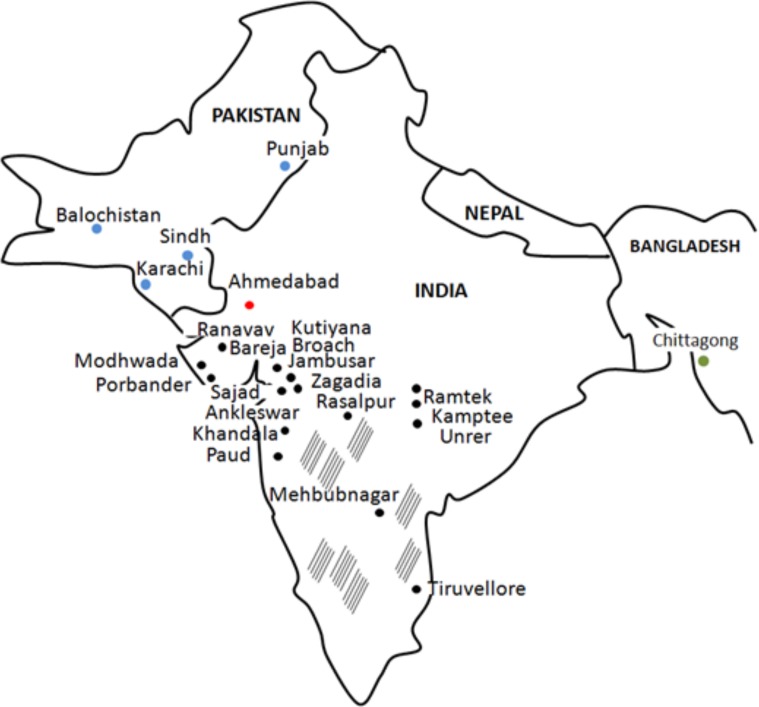
ZIKV cases in India (1954 and 2016 years), Pakistan (1983 year) and Bangladesh (2016 year). Black, India sites from 1954 study; Red, India sites from 2016 study; Blue, Pakistan sites from 1983 study; Green, Bangladesh from 2016 study; striped areas, sites tested negative for ZIKV antibody in 1954 study.

It is believed that ZIKV reached Asia in 1970s, when cases were documented in Pakistan and Indonesia (Olson et al., 1981; Darwish et al., 1983). However, a research study by Smithburn et al. (1954) suggests that ZIKV was already circulating in the region as ZIKV neutralizing antibodies were detected in healthy donor serum in 1952. This study utilized serum samples collected in 1952, which is 5 years after isolation of ZIKV in Uganda, Africa (Dick et al., 1952). These findings bring up a possibility of ZIKV spreading far distance from the site of origin before it was even first isolated in Africa. In 1954 study of ZIKV antibody prevalence in Indian population, a total of 588 serum samples were collected from donors residing in 38 widely scattered locations (Smithburn et al., 1954; **Figure 1**), while 196 samples were tested for presence of anti-ZIKV neutralizing antibodies. Selection of the sites for the blood collection was made to cover the large geographic area as well as to include locations with high rainfall or forest, which could affect the epidemiology of arthropod-borne virus infection. To ensure that seroprevalence is strictly associated with local infection, individuals traveling any great distances were excluded. Neutralizing antibodies were detected in 33 serum samples out of 196 tested (16.8%). ZIKV seropositive donors were detected in many locations, including those near Bombay (Broach district; 7 of 17 immune), Saurashtra (Sorath district; 4 of 11 protective), Madhya Pradesh (Nagpur district; 7 of 15 protective), Hyderabad and Madras (**Figure 1**). It appears that there are several locations with high seropositivity rate including Bareja and Nagpur. Interestingly, authors state that the single site showing the highest rate of seropositivity was Bareja, which is located in the Ahmedabad district. The same site, the Ahmedabad city, is where ZIKV positive samples were recently reported by Sapkal et al. (2018). Therefore, it could be suggested that ZIKV was circulating in this region for more than 6 decades, without association with major outbreaks. It was an interesting observation that presence of ZIKV neutralizing antibodies was not associated with protection against any other viruses included in the study by Smithburn et al. (Dick et al., 1952). Therefore, authors conclude that seropositivity is most likely associated with infection with ZIKV and is not a result of cross-reactivity.

## ZIKV Genotype in India

First ZIKV strain was isolated in 1947 from sentinel monkey in the Zika forest in Uganda, Africa (Dick et al., 1952). Only sporadic cases of ZIKV infection were reported in following 6 decades (Pettersson et al., 2016). Two ZIKV outbreaks, in 2007 and 2013, announced the new era in virus epidemiology (Duffy et al., 2009; Roth et al., 2014). These outbreaks are characterized by a large number of symptomatic cases and rapid spread of ZIKV across the Pacific Ocean islands reaching South America (Roth et al., 2014; Dupont-Rouzeyrol et al., 2015; Tognarelli et al., 2016). Genetic analysis revealed that ZIKV evolved rapidly undergoing multiple nucleotide changes in viral RNA have accumulated over time between 1947 and 2007 (Wang et al., 2017). Interestingly, it appears that numerous changes in virus genome occurred during the period of “epidemiological silence,” when only a few cases of ZIKV infection were documented around the world. Many new strains were identified, comprising two major lineages: African and Asian (including South American) (Gong et al., 2016). The Asian lineage prototype was isolated from *A. aegypti* mosquitoes collected in Malaysia in 1966 (Marchette et al., 1969; Wang et al., 2017), and it is the lineage currently associated with microcephaly (Saiz et al., 2016).

Zika virus sequences obtained from Ahmedabad, India, cases revealed their close relationship to Asian strains (Sapkal et al., 2018). Interestingly, there is a possible connection between Indian cases from 2017 and ZIKV seropositivity reports from Pakistan in 1983 (Darwish et al., 1983). In a study of Darwish et al. ZIKV antibody was detected in animal and human samples collected from several sites in Pakistan including Karachi, Sind, Baluchistan and Punjab Provinces (Darwish et al., 1983; **Figure 1**). Two sites, Karachi and Punjab province in Pakistan are in close proximity to the Indian border, while Karachi is near Ahmedabad, India. ZIKV’s association with microcephaly and GBS during the recent outbreak prompted investigations on circulation of the virus in Bangladesh, country neighboring India (**Figure 1**). One serum sample was found positive for ZIKV RNA when 200 samples collected at various locations in Bangladesh were analyzed (Muraduzzaman et al., 2017). Phylogenetic analysis revealed that this sample clusters together with “Asian lineage” of ZIKV. Interestingly, the same, “Asian lineage” ZIKV was identified in Ahmedabad, India by Sapkal et al. (2018). ZIKV positive sample was obtained from individual who never traveled outside of the region or internationally, suggesting that infection occurred locally. Taken together, these data indicates that “Asian lineage” ZIKV is the main strain circulating in this region and is associated with human infections.

Zika virus surveillance study in Pakistan revealed that many animals were also seropositive for ZIKV, suggesting that different hosts, besides human, could be involved in ZIKV circulation in this region (Darwish et al., 1983). Also, domestic animals, for e.g., sheep and goat, were also found seropositive for ZIKV. It could be suggested that *Culex* mosquitoes are passing ZIKV to the livestocks, as it has been shown to feed off pigs, sheep and birds. On the contrary, the *Aedes* feed predominantly on humans (24%), cats (21%), dogs (14%), and birds (7%). These observations point out the importance of *Aedes* and *Culex* mosquitoes control.

## Challenges of ZIKV Diagnostics and Treatment

The Asian lineage of ZIKV is characterized by mild form of infection, with symptoms mainly including skin rashes, fever and signs of inflammation. Interestingly, fever of unknown etiology is common diagnosis in the rural hospitals in India and Pakistan. Limited access to clinical diagnostics hampers identification of infectious agent making many ZIKV cases remain undetected. This brings to attention the necessity of simple and low-cost diagnostics for ZIKV infection in the region. These diagnostics should be simple in use and require less complex and expensive equipment. One of the tests which could fit the requirements is the lateral flow immunoassay (LFI), often referred to as “dip stick.” LFI combines unique advantages of bio-recognition probes and chromatography, where results are evaluated visually. Variety of samples could be used for LFI, including, urine, serum, plasma, and saliva (Magambo et al., 2014; Carrio et al., 2015; Moreno et al., 2017). LFI diagnostics have long shelf life, use small quantity of sample and do not require refrigeration, which makes them well adapted for use in rural areas. Multiple strategies were applied to develop LFI based diagnostics (Lee et al., 2016; Balmaseda et al., 2017; Choi et al., 2017), still more research is required to identify the test which would be specific, easy to use and inexpensive.

Zika virus diagnostics is challenging because of extensive antibody cross-reactivity against related flaviviruses, e.g., DENV (Cao-Lormeau et al., 2016). Serum from ZIKV-infected patients and ZIKV/DENV-co-infected individuals reacted with envelope (E) and non-structural 1 (NS1) proteins of ZIKV as well as DENV (Stettler et al., 2016). The E, pre-membrane (prM), and the NS1 proteins are the main antigens associated with targets for human B-cell responses in DENV infections (Priyamvada et al., 2016; Stettler et al., 2016). It is believed that considerable structural and genetic similarities between ZIKV and DENV are responsible for observed antibody cross-reactivity between these closely related viruses. The E protein binds to the receptor triggering viral entry; therefore, it is a major target for neutralizing antibody responses (Sukupolvi-Petty et al., 2007). Cross-reactive antibodies can be protective, as they can interfere with virus binding to the receptor and entering the cell. However, these antibodies could also exacerbate the disease by increasing the rate of infection (Halstead, 2003). This effect was explained by antibody-dependent enhancement (ADE, Russell et al., 2018), where cross-reacting antibodies form immune complexes with the antigen. These complexes are more efficiently taken up by leukocytes *via* Fc-receptors (Halstead, 2003). ADE has been shown between antibodies against DENV and ZIKV (Dejnirattisai et al., 2016; Priyamvada et al., 2016). These studies are important in the view that India is also endemic for DENV, therefore, it could be suggested that cross-reactivity between antibodies to ZIKV and DENV could promote virus spread and contribute to ZIKV epidemics. However, there is still lack of evidence that ADE contributes to ZIKV epidemics (Martin-Acebes et al., 2018). Nanopore based sequencing technology is a novel powerful tool for detection of viruses directly from clinical samples (Quick et al., 2017). This sequencing approach does not require isolation and culturing the virus, yet it provides sufficient number of reads to identify the infectious agent in as small number of copies as 50 genomes per reaction. Data analysis is based on simple to follow protocol and it could be done in remote areas with limited access to the internet for the analysis. The multi-fold genome coverage allows the MinION nanopore sequencing to reach over 99% accuracy of the post-data analysis, which is sufficient for accurate detection of known viruses (Wang et al., 2015). This technology was successfully used for detection of multiple arboviruses including DENV, ZIKV, and Venezuelan Equine Encephalitis Virus (Mohamed et al., 2017; Quick et al., 2017; Russell et al., 2018).

Presently, there is no effective treatment or vaccine for ZIKV infection. Therapy is mainly supportive and includes fluid and pain management. Prevention includes mosquito bite control including personal protection. Caution is advised to pregnant women traveling to ZIKV endemic countries. Testing should be available to those traveling to endemic countries; special attention should be given to pregnant women and individuals with fever and skin rash. It is essential to conduct thorough examination of the fetus for early diagnosis of brain deformities.

## Will ZIKV Microcephaly Ever Be Diagnosed in India?

As reported, ZIKV epidemiology began in Africa, when the first strain of virus, was isolated in Uganda (African lineage; 1947) (Dick et al., 1952). Later, ZIKV seroconversion was documented in Asia (Asian lineage; 1966), followed by outbreaks in Pacific Islands (Asian lineage; 2007) and South America (Asian/American lineage; early 2015) (Plourde and Bloch, 2016). Crossing of the Pacific Ocean and invasion of South America has been associated with dramatic changes in clinical manifestation of ZIKV infection, as for the first time, severe neurological disorders, such as congenital ZIKV syndrome (microcephaly and other developmental defects in neonatal brain) are now linked to ZIKV pathogenesis (de Oliveira et al., 2017). It appears that over years, a lesser-known ZIKV that left Zika forest in Uganda has dramatically evolved from a mild-disease causing virus to potentially a more-virulent and deadly virus. Whether the new contemporary epidemic ZIKV strain belonging to Asian/American lineage will remain in Americas or continue traveling across several countries to finally return to the land of its origin, still remains unclear.

**FIGURE 2:**
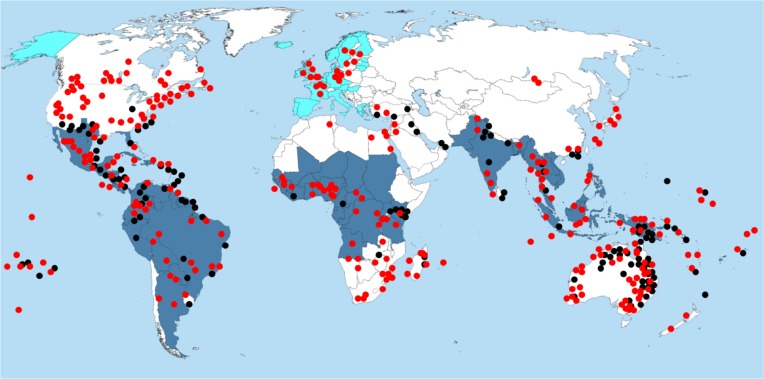
Distribution of Aedes and Culex mosquito species and diagnosis of ZIKV infection. Red dots, distribution of Aedes species; Black dots, distribution of Culex species; Dark blue area, regions where ZIKV infection was diagnosed; Light blue area, regions where travel related ZIKV infection were diagnosed.

In this regards, periodic imported cases of ZIKV infection due to South American strains of ZIKV have already been documented in Europe (Baronti et al., 2014; European Centre for Disease Prevention and Control, 2016; Zander, 2016; Public Health England, 2017; Boyer et al., 2018; **Figure 2**). According to the European Center for Disease Prevention and Control (ECDC), invasive ZIKV-vectors, including *Aedes aegypti* and *Aedes albopictus* have been reported in several European countries, including Austria, Bosnia, France, Germany, and Italy (ECDC, 2016). In addition, though the reported ZIKV imported cases are all travel-associated cases, the *Aedes* mosquitoes in the natural reservoirs could potentially become infected while feeding off the viremic ZIKV-infected individuals and spread the infection across the resident population. It has also been suggested that the climate change as well as human activities can contribute to *Aedes* species spread into the nearby territories and establish new sustainable populations (ECDC, 2016). As these species are most substantial vectors of ZIKV transmission to humans, it could be suggested that requirements for ZIKV outbreak are now present in Europe. Introducing the new contemporary ZIKV Asian/American strain into *Aedes* population in Europe may also cause newborn microcephaly and neurological complications due to ZIKV infection. As Rocklov et al. (2016) has shown, travel from ZIKV endemic regions of the Americas aligns with peak predicted capacity of *Aedes* vectors to transmit infection. Europe is in close proximity to Africa and has close commercial and historic ties with many African countries. Therefore, there is a potential threat that now ZIKV, targeting the fetus brain, can cause outrageous epidemics in Africa, and subsequently spread throughout Asia. In addition, due to a combination of factors, including visitors infected with ZIKV, weak cross-border surveillance, climate change, and lack of effective vector control measures, India and neighboring countries (Pakistan and Bangladesh) are pre-disposed to ZIKV importation and epidemics in the near future, causing a potential public health concern (Bogoch et al., 2016). A close global monitoring of ZIKV epidemiology is therefore required to predict ZIKV outbreaks caused by new contemporary virulent ZIKV strain.

## Author Contributions

SK conceived of the original idea and wrote the sections-Introduction and Modes of ZIKV transmission. TU wrote the sections-Historical overview of ZIKV in India and ZIKV genotype in India. EM prepared all the original figures. MB wrote the section-Challenges of ZIKV diagnostics and treatment. AR wrote the section-Will ZIKV microcephaly ever be diagnosed in India. SV did the overall supervision, editing, and managing of the multisite collaborations.

## Conflict of Interest Statement

The authors declare that the research was conducted in the absence of any commercial or financial relationships that could be construed as a potential conflict of interest.
